# 3-Carbamoyl-2,2-dimethyl­cyclo­pentane-1,1-dicarb­oxy­lic acid

**DOI:** 10.1107/S1600536812005636

**Published:** 2012-02-17

**Authors:** Volodymyr Knizhnikov, Marian Gorichko, Zoya Voitenko

**Affiliations:** a National Taras Shevchenko University, Department of Chemistry, Volodymyrska Street 64, 01033 Kyiv, Ukraine

## Abstract

In the title compound, C_10_H_15_NO_5_, the five-membered cyclo­pentane ring has an envelope conformation, with four atoms lying in a plane (mean deviation = 0.0213 Å), while the fifth atom deviates from this plane by 0.626 (2) Å. A three-dimensional structure is formed through N—H⋯O and O—H⋯O hydrogen bonds between the amide and carb­oxy­lic acid groups and both carb­oxy­lic acid and amide O-atom acceptors.

## Related literature

For background literature, see: Carter (1958 ▶); Nieto *et al.* (1998 ▶); Noyes (1894 ▶). For the synthetic procedure, see: Polonski (1982 ▶, 1983 ▶).
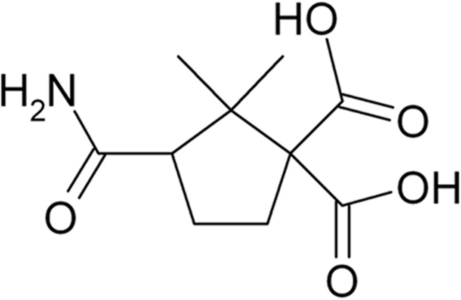


## Experimental

### 

#### Crystal data

C_10_H_15_NO_5_
*M* *_r_* = 229.23
Tetragonal, 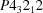

*a* = 9.4424 (1) Å
*c* = 24.7343 (5) Å
*V* = 2205.28 (6) Å^3^
*Z* = 8
Mo *K*α radiation
μ = 0.11 mm^−1^
*T* = 296 K
0.36 × 0.20 × 0.19 mm

#### Data collection

Bruker SMART APEXII area-detector diffractometer
Absorption correction: multi-scan (*SADABS*; Bruker, 2008 ▶) *T*_min_ = 0.961, *T*_max_ = 0.979
17322 measured reflections
2917 independent reflections
2602 reflections with *I* > 2σ(*I*)
*R*_int_ = 0.036

#### Refinement

*R*[*F* ^2^ > 2σ(*F* ^2^)] = 0.037
*wR*(*F* ^2^) = 0.099
*S* = 1.06
2917 reflections
163 parameters
H atoms treated by a mixture of independent and constrained refinement
Δρ_max_ = 0.25 e Å^−3^
Δρ_min_ = −0.19 e Å^−3^

### 

Data collection: *APEX2* (Bruker, 2007 ▶); cell refinement: *SAINT* (Bruker, 2007 ▶); data reduction: *SAINT*; program(s) used to solve structure: *SHELXS97* (Sheldrick, 2008 ▶); program(s) used to refine structure: *SHELXL97* (Sheldrick, 2008 ▶); molecular graphics: *SHELXTL* (Sheldrick, 2008 ▶); software used to prepare material for publication: *SHELXTL*.

## Supplementary Material

Crystal structure: contains datablock(s) I, global. DOI: 10.1107/S1600536812005636/zs2175sup1.cif

Structure factors: contains datablock(s) I. DOI: 10.1107/S1600536812005636/zs2175Isup2.hkl

Supplementary material file. DOI: 10.1107/S1600536812005636/zs2175Isup3.cml

Additional supplementary materials:  crystallographic information; 3D view; checkCIF report

## Figures and Tables

**Table 1:** Hydrogen-bond geometry (Å, °)

*D*—H⋯*A*	*D*—H	H⋯*A*	*D*⋯*A*	*D*—H⋯*A*
N1—H15⋯O2^i^	0.869 (19)	2.23 (2)	3.0474 (15)	157.6 (18)
N1—H16⋯O4^ii^	0.89 (2)	2.09 (2)	2.9610 (17)	166.2 (19)
O3—H17⋯O1^iii^	0.76 (2)	1.91 (2)	2.6691 (15)	174 (2)
O5—H18⋯O1^iv^	0.87 (2)	1.80 (2)	2.6569 (14)	168 (2)

Symmetry codes: (i) 
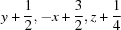
; (ii) 
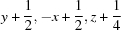
; (iii) 
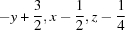
; (iv) 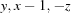.

**Fig. 1.**
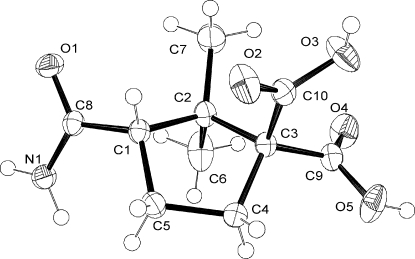
The molecular structure and atom numbering scheme for the title compound, showing 50% probability displacement ellipsoids.

**Fig. 2.**
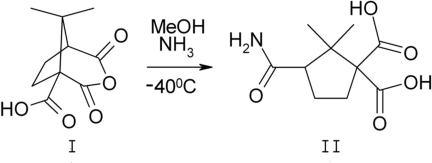
The synthetic route to the title compound (II).

## supplementary crystallographic information

### Comment

Camphoric acids and their derivatives, especially those with specific absolute configurations, are very useful intermediates in organic synthesis (Nieto *et al.*, 1998). Molecules bearing camphoric acid moieties could be used as building blocks in self-assembly studies *via* coordinative and hydrogen bonds leading to network materials with interesting topologies and functions. Herein, we report the synthesis and crystal structure of the title compound (II), the novel 3-(aminocarbonyl)-2,2-dimethylcyclopentane-1,1-dicarboxylic acid, C_10_H_15_NO_5_ (Fig. 1) obtained as a minor product in the ring-opening reaction of 8,8-dimethyl-2,4-dioxo-3-oxabicyclo[3.2.1]octane-1-carboxylic acid (I) (Polonski, 1983) (see Fig. 2).

In the structure of (II) (Fig. 1), the five-membered C1—C5 ring has an envelope conformation, which is typical for this class of compounds. The C1—C3—C4—C5 atoms lie in a plane (mean deviation, 0.0213 Å) while C2 deviates from this plane by 0.626 (2) Å. The bond lengths C1—C2 and C2—C3 [1.5707 (16) and 1.5752 (17) Å respectively] are somewhat longer than the normal single Csp^3^—Csp^3^ bond length. Other C—C bond lengths observed in this compound are unremarkable and fall in the range of 1.5285 (19)–1.5489 (16) Å. A three-dimensional network structure is formed through intermolecular N—H···O hydrogen bonds between the amide and carboxyl groups and O—H···O hydrogen bonds between the carboxylic acid groups and amide O-atom acceptors (Table 1).

### Experimental

The synthesis of the cyclic anhydride (I) (Fig. 2) was carried out according to the method described by Polonski (1983). Compound (I) (1.00 g, 4.36 mmol) was added in three portions to the cooled (-40 °C) saturated solution of ammonia in methanol (10 ml). The resulting solution was stirred for 1 h and white needles formed during this period were filtrated off. These needles can be easily dissolved in water. The mother liquor was acidified with dilute hydrochloric acid to pH 3 and allowed to stand for 24 h. The resulting white needles of the title compound were collected by filtration. Yield: 200 mg, 18.5%; m.p. 237–238 °C. 1H NMR (400 MHz, [D6]DMSO, TMS, δ): 0.81 (s, 3 H), 1.31 (s, 3 H), 1.62–1.71 (m, 1 H), 1.88–1.95 (m, 1H), 1.97–2.07 (m, 1 H), 2.25–2.33 (m, 1 H), 2.98 (t, 3 J = 9.6 Hz, 1 H), 6.84 (s, 1 H), 7.18 (s, 1 H), 12.63 (br. s, 2 H); 13C{1H} NMR (100.70 MHz, [D6]DMSO, TMS, δ): 20.8, 23.1, 23.1, 30.4, 46.2, 52.9, 67.1, 171.5, 173.2, 173.6; (KBr plates, cm -1): 3421, 3328, 3254, 3008, 2976, 2941, 2777, 2595, 1737, 1721, 1651, 1551, 1263, 1242, 1207, 637.

### Refinement

Carboxylic acid and amide H atoms were located in a difference Fourier synthesis and both positional and displacement parameters were allowed to refine. Other H atoms were positioned geometrically, with C—H = 0.96–0.98 Å and were allowed to ride on their parent atoms, with *U*_iso_(H) = 1.2*U*_eq_(methine or methylene C) or 1.5*U*_eq_(methyl C). In the absence of a suitable heavy atom, the absolute configuration of the title compound could not be determined (1146 Friedel pairs).

### Crystal data

**Table:**

C_10_H_15_NO_5_	*D*_x_ = 1.381 Mg m^−^^3^
*M**_r_* = 229.23	Mo *K*α radiation, λ = 0.71073 Å
Tetragonal, *P*4_3_2_1_2	Cell parameters from 6074 reflections
*a* = 9.4424 (1) Å	θ = 2.3–28.8°
*c* = 24.7343 (5) Å	µ = 0.11 mm^−^^1^
*V* = 2205.28 (6) Å^3^	*T* = 296 K
*Z* = 8	Block, colourless
*F*(000) = 976	0.36 × 0.20 × 0.19 mm

### Data collection

**Table:**

Siemens SMART CCD area-detector diffractometer	2917 independent reflections
Radiation source: fine-focus sealed tube	2602 reflections with *I* > 2σ(*I*)
Graphite monochromator	*R*_int_ = 0.036
ω scans	θ_max_ = 29.0°, θ_min_ = 2.3°
Absorption correction: multi-scan (*SADABS*; Bruker, 2008)	*h* = −12→12
*T*_min_ = 0.961, *T*_max_ = 0.979	*k* = −8→12
17322 measured reflections	*l* = −33→33

### Refinement

**Table:**

Refinement on *F*^2^	Primary atom site location: structure-invariant direct methods
Least-squares matrix: full	Secondary atom site location: difference Fourier map
*R*[*F*^2^ > 2σ(*F*^2^)] = 0.037	Hydrogen site location: difference Fourier map
*wR*(*F*^2^) = 0.099	H atoms treated by a mixture of independent and constrained refinement
*S* = 1.06	*w* = 1/[σ^2^(*F*_o_^2^) + (0.0615*P*)^2^ + 0.0801*P*] where *P* = (*F*_o_^2^ + 2*F*_c_^2^)/3
2917 reflections	(Δ/σ)_max_ < 0.001
163 parameters	Δρ_max_ = 0.25 e Å^−^^3^
0 restraints	Δρ_min_ = −0.19 e Å^−^^3^

### Special details

**Table:**

Geometry. All esds (except the esd in the dihedral angle between two l.s. planes) are estimated using the full covariance matrix. The cell esds are taken into account individually in the estimation of esds in distances, angles and torsion angles; correlations between esds in cell parameters are only used when they are defined by crystal symmetry. An approximate (isotropic) treatment of cell esds is used for estimating esds involving l.s. planes.
Refinement. Refinement of *F*^2^ against ALL reflections. The weighted *R*-factor *wR* and goodness of fit *S* are based on *F*^2^, conventional *R*-factors *R* are based on *F*, with *F* set to zero for negative *F*^2^. The threshold expression of *F*^2^ > σ(*F*^2^) is used only for calculating *R*-factors(gt) *etc*. and is not relevant to the choice of reflections for refinement. *R*-factors based on *F*^2^ are statistically about twice as large as those based on *F*, and *R*-factors based on ALL data will be even larger.

### Fractional atomic coordinates and isotropic or equivalent isotropic displacement parameters (Å^2^)

**Table:**

	*x*	*y*	*z*	*U*_iso_*/*U*_eq_	
C1	0.74432 (13)	0.23875 (13)	0.07554 (4)	0.0200 (2)	
H1	0.8314	0.2766	0.0595	0.024*	
C2	0.63594 (14)	0.21880 (14)	0.02794 (5)	0.0224 (3)	
C3	0.69569 (13)	0.07984 (13)	0.00103 (5)	0.0193 (2)	
C4	0.74117 (16)	−0.01308 (14)	0.04958 (5)	0.0253 (3)	
H4A	0.6643	−0.0751	0.0604	0.030*	
H4B	0.8224	−0.0708	0.0400	0.030*	
C5	0.77903 (16)	0.08881 (15)	0.09546 (5)	0.0263 (3)	
H5A	0.8789	0.0813	0.1043	0.032*	
H5B	0.7243	0.0667	0.1276	0.032*	
C6	0.48721 (16)	0.1928 (2)	0.05104 (6)	0.0395 (4)	
H6A	0.4565	0.2753	0.0705	0.059*	
H6B	0.4897	0.1131	0.0751	0.059*	
H6C	0.4225	0.1739	0.0220	0.059*	
C7	0.6348 (2)	0.34634 (17)	−0.00998 (6)	0.0383 (4)	
H7A	0.5754	0.3264	−0.0406	0.057*	
H7B	0.7295	0.3653	−0.0221	0.057*	
H7C	0.5988	0.4275	0.0089	0.057*	
C8	0.69559 (14)	0.34575 (14)	0.11739 (5)	0.0215 (3)	
C9	0.59168 (14)	0.00109 (15)	−0.03551 (5)	0.0244 (3)	
C10	0.82601 (14)	0.11865 (14)	−0.03276 (5)	0.0224 (3)	
N1	0.64079 (14)	0.30205 (14)	0.16346 (4)	0.0266 (3)	
O1	0.70824 (12)	0.47490 (10)	0.10779 (3)	0.0319 (3)	
O2	0.93895 (11)	0.14705 (13)	−0.01307 (4)	0.0359 (3)	
O3	0.80177 (13)	0.12032 (15)	−0.08521 (4)	0.0412 (3)	
O4	0.48796 (12)	0.05118 (13)	−0.05648 (4)	0.0394 (3)	
O5	0.62860 (13)	−0.13224 (12)	−0.04156 (5)	0.0401 (3)	
H15	0.618 (2)	0.369 (2)	0.1859 (7)	0.040 (5)*	
H16	0.628 (2)	0.211 (2)	0.1705 (8)	0.045 (5)*	
H17	0.869 (2)	0.144 (2)	−0.0996 (9)	0.054 (6)*	
H18	0.568 (3)	−0.176 (3)	−0.0621 (8)	0.062 (6)*	

### Atomic displacement parameters (Å^2^)

**Table:**

	*U*^11^	*U*^22^	*U*^33^	*U*^12^	*U*^13^	*U*^23^
C1	0.0188 (6)	0.0222 (6)	0.0189 (5)	0.0011 (5)	0.0020 (4)	−0.0030 (4)
C2	0.0204 (6)	0.0241 (6)	0.0228 (5)	0.0050 (5)	−0.0013 (5)	−0.0039 (5)
C3	0.0172 (6)	0.0216 (6)	0.0191 (5)	0.0013 (5)	0.0001 (4)	−0.0025 (4)
C4	0.0321 (7)	0.0211 (6)	0.0227 (5)	0.0000 (6)	−0.0030 (5)	0.0005 (4)
C5	0.0322 (8)	0.0250 (7)	0.0216 (5)	0.0059 (6)	−0.0030 (5)	−0.0014 (5)
C6	0.0172 (7)	0.0601 (11)	0.0413 (7)	0.0031 (7)	0.0031 (6)	−0.0200 (7)
C7	0.0556 (10)	0.0277 (7)	0.0316 (7)	0.0139 (7)	−0.0111 (7)	0.0006 (6)
C8	0.0202 (6)	0.0232 (6)	0.0213 (5)	−0.0012 (5)	0.0015 (4)	−0.0042 (5)
C9	0.0213 (6)	0.0295 (7)	0.0225 (5)	−0.0019 (5)	0.0010 (5)	−0.0042 (5)
C10	0.0210 (6)	0.0241 (6)	0.0221 (5)	0.0035 (5)	0.0031 (5)	−0.0020 (5)
N1	0.0347 (7)	0.0232 (6)	0.0221 (5)	−0.0004 (5)	0.0082 (5)	−0.0030 (4)
O1	0.0462 (6)	0.0207 (5)	0.0289 (5)	−0.0035 (4)	0.0146 (4)	−0.0027 (4)
O2	0.0201 (5)	0.0592 (7)	0.0286 (5)	−0.0042 (5)	0.0023 (4)	−0.0063 (5)
O3	0.0310 (6)	0.0710 (9)	0.0214 (5)	−0.0108 (6)	0.0030 (4)	0.0042 (5)
O4	0.0319 (6)	0.0432 (7)	0.0429 (6)	0.0057 (5)	−0.0169 (5)	−0.0058 (5)
O5	0.0369 (6)	0.0322 (6)	0.0512 (6)	0.0027 (5)	−0.0174 (5)	−0.0171 (5)

### Geometric parameters (Å, º)

**Table:**

C1—C8	1.5180 (16)	C6—H6B	0.9600
C1—C5	1.5344 (17)	C6—H6C	0.9600
C1—C2	1.5713 (16)	C7—H7A	0.9600
C1—H1	0.9800	C7—H7B	0.9600
C2—C7	1.5265 (19)	C7—H7C	0.9600
C2—C6	1.536 (2)	C8—O1	1.2481 (16)
C2—C3	1.5757 (17)	C8—N1	1.3177 (16)
C3—C9	1.5279 (18)	C9—O4	1.2049 (17)
C3—C10	1.5319 (18)	C9—O5	1.3149 (18)
C3—C4	1.5480 (16)	C10—O2	1.2026 (17)
C4—C5	1.5301 (17)	C10—O3	1.3175 (16)
C4—H4A	0.9700	N1—H15	0.869 (19)
C4—H4B	0.9700	N1—H16	0.89 (2)
C5—H5A	0.9700	O3—H17	0.76 (2)
C5—H5B	0.9700	O5—H18	0.87 (2)
C6—H6A	0.9600		
			
C8—C1—C5	117.38 (10)	C1—C5—H5B	110.3
C8—C1—C2	113.16 (10)	H5A—C5—H5B	108.6
C5—C1—C2	105.61 (10)	C2—C6—H6A	109.5
C8—C1—H1	106.7	C2—C6—H6B	109.5
C5—C1—H1	106.7	H6A—C6—H6B	109.5
C2—C1—H1	106.7	C2—C6—H6C	109.5
C7—C2—C6	110.36 (13)	H6A—C6—H6C	109.5
C7—C2—C1	111.75 (11)	H6B—C6—H6C	109.5
C6—C2—C1	109.63 (10)	C2—C7—H7A	109.5
C7—C2—C3	113.57 (10)	C2—C7—H7B	109.5
C6—C2—C3	110.58 (12)	H7A—C7—H7B	109.5
C1—C2—C3	100.55 (9)	C2—C7—H7C	109.5
C9—C3—C10	108.06 (10)	H7A—C7—H7C	109.5
C9—C3—C4	111.18 (11)	H7B—C7—H7C	109.5
C10—C3—C4	109.63 (11)	O1—C8—N1	120.54 (12)
C9—C3—C2	115.15 (10)	O1—C8—C1	119.44 (11)
C10—C3—C2	108.59 (10)	N1—C8—C1	120.02 (12)
C4—C3—C2	104.10 (9)	O4—C9—O5	122.84 (12)
C5—C4—C3	106.48 (10)	O4—C9—C3	125.88 (13)
C5—C4—H4A	110.4	O5—C9—C3	111.28 (11)
C3—C4—H4A	110.4	O2—C10—O3	123.37 (12)
C5—C4—H4B	110.4	O2—C10—C3	123.01 (11)
C3—C4—H4B	110.4	O3—C10—C3	113.61 (11)
H4A—C4—H4B	108.6	C8—N1—H15	115.0 (12)
C4—C5—C1	106.98 (10)	C8—N1—H16	121.8 (13)
C4—C5—H5A	110.3	H15—N1—H16	123.2 (18)
C1—C5—H5A	110.3	C10—O3—H17	108.7 (17)
C4—C5—H5B	110.3	C9—O5—H18	110.7 (17)
			
C8—C1—C2—C7	72.70 (14)	C8—C1—C5—C4	147.86 (12)
C5—C1—C2—C7	−157.59 (11)	C2—C1—C5—C4	20.66 (14)
C8—C1—C2—C6	−49.99 (15)	C5—C1—C8—O1	157.82 (13)
C5—C1—C2—C6	79.72 (14)	C2—C1—C8—O1	−78.73 (15)
C8—C1—C2—C3	−166.49 (10)	C5—C1—C8—N1	−22.01 (18)
C5—C1—C2—C3	−36.77 (12)	C2—C1—C8—N1	101.44 (14)
C7—C2—C3—C9	−79.30 (14)	C10—C3—C9—O4	−100.66 (15)
C6—C2—C3—C9	45.41 (15)	C4—C3—C9—O4	138.99 (14)
C1—C2—C3—C9	161.20 (10)	C2—C3—C9—O4	20.92 (19)
C7—C2—C3—C10	41.99 (15)	C10—C3—C9—O5	79.70 (14)
C6—C2—C3—C10	166.70 (10)	C4—C3—C9—O5	−40.65 (15)
C1—C2—C3—C10	−77.51 (11)	C2—C3—C9—O5	−158.73 (11)
C7—C2—C3—C4	158.73 (12)	C9—C3—C10—O2	−159.99 (13)
C6—C2—C3—C4	−76.56 (13)	C4—C3—C10—O2	−38.67 (17)
C1—C2—C3—C4	39.23 (12)	C2—C3—C10—O2	74.46 (16)
C9—C3—C4—C5	−152.41 (11)	C9—C3—C10—O3	21.14 (16)
C10—C3—C4—C5	88.17 (12)	C4—C3—C10—O3	142.46 (12)
C2—C3—C4—C5	−27.84 (14)	C2—C3—C10—O3	−104.41 (13)
C3—C4—C5—C1	4.56 (15)		

### Hydrogen-bond geometry (Å, º)

**Table:**

*D*—H···*A*	*D*—H	H···*A*	*D*···*A*	*D*—H···*A*
N1—H15···O2^i^	0.869 (19)	2.23 (2)	3.0474 (15)	157.6 (18)
N1—H16···O4^ii^	0.89 (2)	2.09 (2)	2.9610 (17)	166.2 (19)
O3—H17···O1^iii^	0.76 (2)	1.91 (2)	2.6691 (15)	174 (2)
O5—H18···O1^iv^	0.87 (2)	1.80 (2)	2.6569 (14)	168 (2)

Symmetry codes: (i) *y*+1/2, −*x*+3/2, *z*+1/4; (ii) *y*+1/2, −*x*+1/2, *z*+1/4; (iii) −*y*+3/2, *x*−1/2, *z*−1/4; (iv) *y*, *x*−1, −*z*.
